# Supplementary material for: Effects of Broad Bean Diet on the Growth Performance, Muscle Characteristics, Antioxidant Capacity, and Intestinal Health of Nile Tilapia (Oreochromis niloticus)
Source: Animals (Basel). 2023 Nov 29;13(23):3705. doi: 10.3390/ani13233705 (PMC10705166; doi:10.3390/ani13233705)
Supplement: Supplementary file 1 [file animals-13-03705-s001.zip › animals-2686003-supplementary.pdf]

Table S1. Amplified primer sequence

Amplified Region	Primer sequence
16S rDNA V3-V4(338-806)	ACTCCTACGGGAGGCAGCAG
	GGACTACHVGGGTWTCTAAT

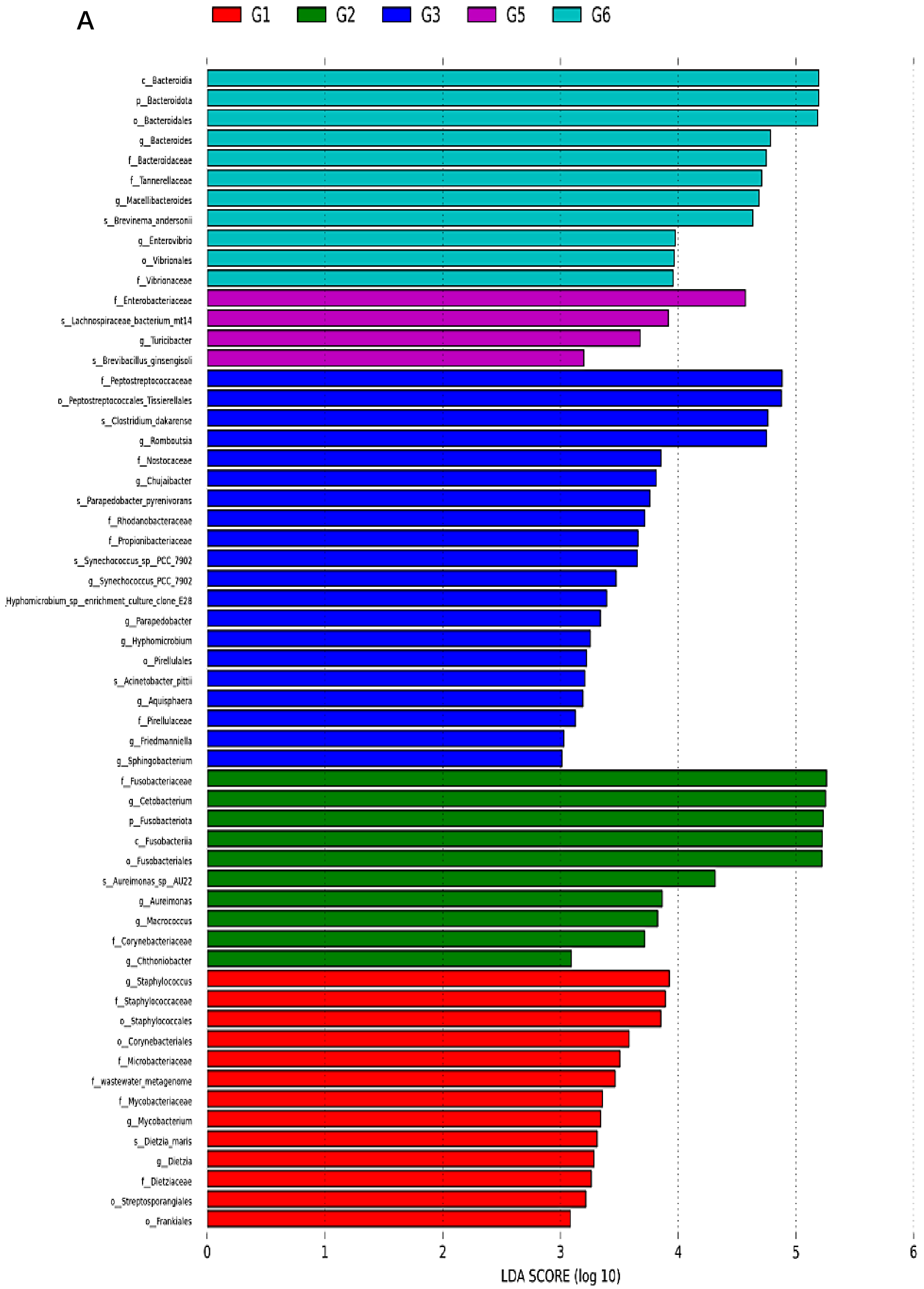
A
G1
G2
G3
G5
G6
c__Bacteroidia
p__Bacteroidota
o__Bacteroidales
g__Bacteroides
f__Bacteroidaceae
f__Tannerellaceae
g__Macellibacteroides
s__Brevinema_andersonii
g__Enterovibrio
o__Vibrionales
f__Vibrionaceae
f__Enterobacteriaceae
s__Lachnospiraceae_bacterium_mt14
g__Turicibacter
s__Brevibacillus_ginsengisoli
f__Peptostreptococcaceae
o__Peptostreptococcales_Tissierellales
s__Clostridium_dakarense
g__Romboutsia
f__Nostocaceae
g__Chujaibacter
s__Parapedobacter_pyrenivorans
f__Rhodanobacteraceae
f__Propionibacteriaceae
s__Synechococcus_sp__PCC_7902
g__Synechococcus_PCC_7902
Hyphomicrobium_sp__enrichment_culture_clone_E28
g__Parapedobacter
g__Hyphomicrobium
o__Pirellulales
s__Acinetobacter_pittii
g__Aquisphaera
f__Pirellulaceae
g__Friedmanniella
g__Sphingobacterium
f__Fusobacteriaceae
g__Cetobacterium
p__Fusobacteriota
c__Fusobacteriia
o__Fusobacteriales
s__Aureimonas_sp__AU22
g__Aureimonas
g__Macrococcus
f__Corynebacteriaceae
g__Chthoniobacter
g__Staphylococcus
f__Staphylococcaceae
o__Staphylococcales
o__Corynebacteriales
f__Microbacteriaceae
f__wastewater_metagenome
f__Mycobacteriaceae
g__Mycobacterium
s__Dietzia_maris
g__Dietzia
f__Dietziaceae
o__Streptosporangiales
o__Frankiales
0
1
2
3
4
5
6
LDA SCORE (log 10)

B

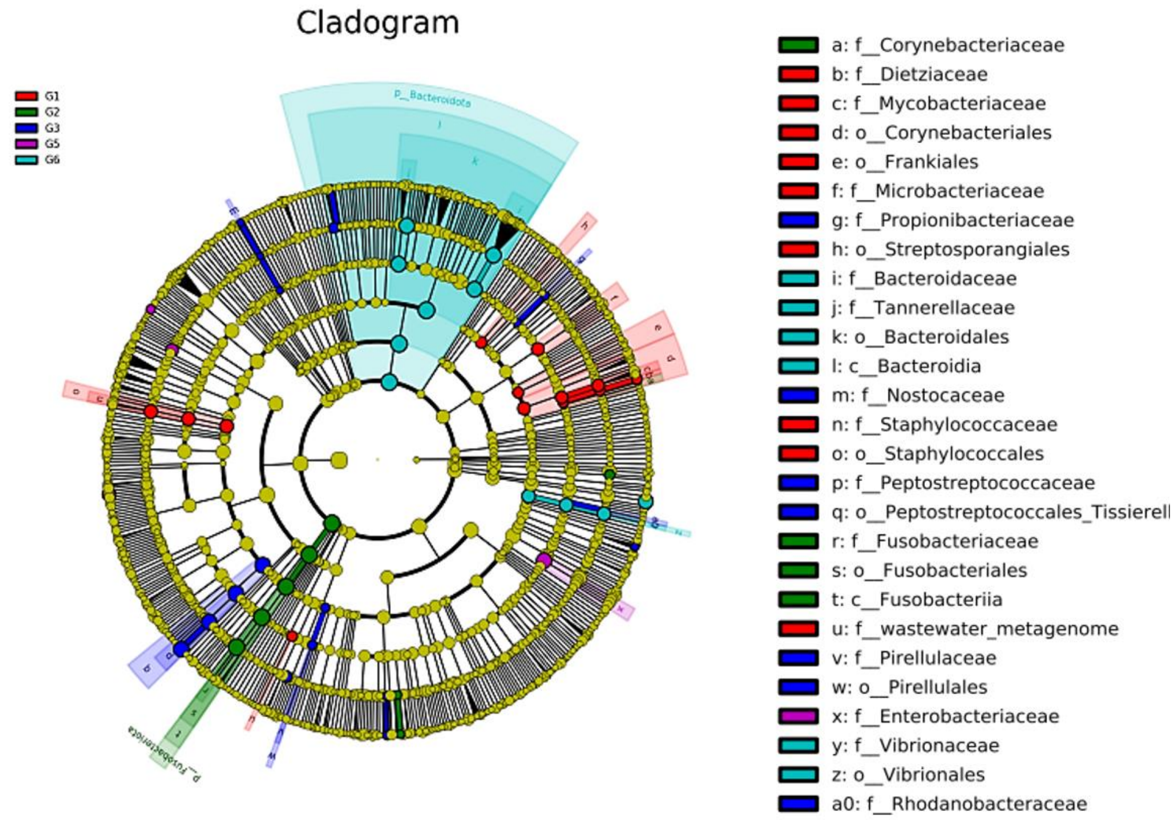

Figure S1. LEfSe analysis based on category information. A: Histogram of LDA distribution based on LEfSe analysis of classification information; B: Evolutionary branching diagram of LEfSe analysis based on taxonomic information. The formulation and nutrient composition of experimental diets G1–G6 please refer to Table 1.

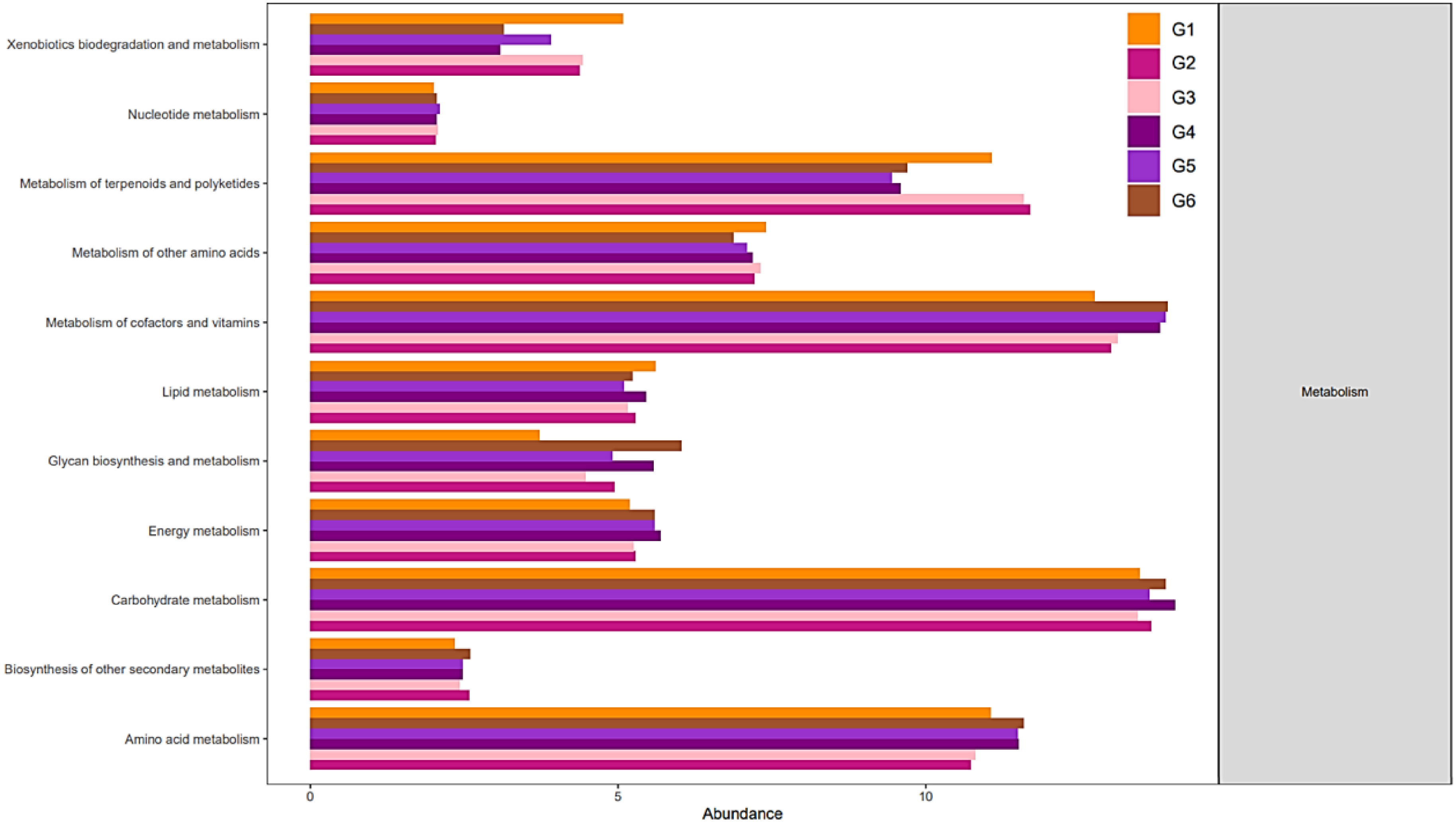

Figure S2. Intestinal microbial community function prediction of Nile tilapia (*Orechromis niloticus*) fed different experimental diets on the second level in metabolism function. There was no significant impact on the first level of the KEGG pathway between groups.
